# The *Chlamydia muridarum* plasmid revisited : new insights into growth kinetics

**DOI:** 10.12688/wellcomeopenres.13905.1

**Published:** 2018-03-08

**Authors:** Rachel J. Skilton, Yibing Wang, Colette O'Neill, Simone Filardo, Peter Marsh, Angèle Bénard, Nicholas R. Thomson, Kyle H. Ramsey, Ian N. Clarke

**Affiliations:** 1 Molecular Microbiology Group, Faculty of Medicine, University of Southampton, Southampton General Hospital, Southampton, UK; 2 Division of Allergy and Infectious Diseases, Department of Medicine, University of Washington, Seattle, WA, USA; 3 Department of Public Health and Infectious Diseases, Section of Microbiology, Sapienza University, Rome, Italy; 4 Public Health England, Public Health Laboratory Southampton, Southampton General Hospital, Southampton, UK; 5 Wellcome Trust Sanger Institute, Wellcome Trust Genome Campus, Hinxton, UK; 6 London School of Hygiene and Tropical Medicine, London, UK; 7 Department of Microbiology & Immunology, Chicago College of Osteopathic Medicine, Midwestern University, Downers Grove, IL, USA

**Keywords:** Chlamydia muridarum, plasmid, phenotype, inclusion, transformation, Nigg

## Abstract

**Background:** Research in chlamydial genetics is challenging because of its obligate intracellular developmental cycle. *In vivo *systems exist that allow studies of different aspects of basic biology of chlamydiae, the murine *Chlamydia muridarum* model is one of great importance and thus an essential research tool. *C. muridarum* carries a plasmid that has a role in virulence.  Our aim was to compare and contrast the *C. muridarum *plasmid-free phenotype with that of a chromosomally isogenic plasmid-bearing strain, through the inclusion phase of the developmental cycle.

**Methods:** We measured infectivity for plasmid bearing and plasmid-cured *C. muridarum* by inclusion forming assays in McCoy cells and in parallel bacterial chromosome replication by quantitative PCR, throughout the developmental cycle. In addition to these studies, we have carefully monitored chlamydial inclusion formation by confocal microscopy and transmission electron microscopy. A new *E.coli*/chlamydial shuttle vector (pNigg::GFP) was constructed using standard cloning technology and used to transform *C. muridarum* for further phenotypic studies.

**Results:** We have advanced the definition of the chlamydial phenotype away from the simple static observation of mature inclusions and redefined the *C. muridarum* plasmid-based phenotype on growth profile and inclusion morphology. Our observations on the growth properties of plasmid-cured *C. muridarum* challenge the established interpretations, especially with regard to inclusion growth kinetics. Introduction of the shuttle plasmid pNigg::GFP into plasmid-cured *C. muridarum* restored the wild-type plasmid-bearing phenotype and confirmed that loss of the plasmid was the sole cause for the changes in growth and chromosomal replication.

**Conclusions:** Accurate growth curves and sampling at multiple time points throughout the developmental cycle is necessary to define plasmid phenotypes.  There are subtle but important (previously unnoticed) differences in the overall growth profile of plasmid-bearing and plasmid-free *C. muridarum*.  We have proven that the differences described are solely due to the plasmid pNigg.

## Introduction

*Chlamydia muridarum* is an obligate intracellular bacterial pathogen. It is used to model the pathogenesis of chlamydial infections in mice ^1^. *C. muridarum* has a genome comprising a single circular chromosome (~1Mb) and a relatively small plasmid of 7.5 kb ^2^. *Chlamydiae* have a unique bi-phasic developmental cycle alternating between the infectious, extracellular, elementary body (EB) and the replicating, non-infectious intracellular form, the reticulate body (RB) ^3^. RBs divide by binary fission within an intracytoplasmic structure known as an ‘inclusion’ ^4^.

Recent discoveries in chlamydial genetics have brought significant advances in the field ^5^ and the biology of the chlamydial plasmid is being unravelled ^6^. *C. muridarum* was the first chlamydial species that was cured of its plasmid artificially ^7^. Inclusions formed by plasmid-free *C. muridarum* display a distinctive morphological phenotype *in vitro.* This phenotype is most evident in near-mature inclusions which, when viewed by phase contrast microscopy, appear to have a translucent centre, the “bull’s eye” inclusion ^7^. In addition, unlike plasmid-bearing isolates, inclusions from plasmid-free isolates of *C. muridarum* and *C. trachomatis* do not stain with iodine, thus they are thought to be unable to accumulate glycogen ^7– 9^. Plasmid-free *Chlamydia* strains are important tools because research progress in chlamydial plasmid genetics is complicated by the presence of the endogenous plasmid. In fact the transforming *Chlamydia / E.coli* plasmid-based shuttle vectors that are derived from endogenous plasmids can recombine with the native plasmid ^10^. The use of isogenic, plasmid-cured chlamydial isolates as recipient hosts alleviates the uncertainty of potential plasmid/vector recombination. Since the chlamydial plasmid is not essential for survival, subsequent *in vitro* genetic studies, mainly in *C. trachomatis,* have indicated the role of plasmid-encoded factors in chlamydial biology by analysing the effects of single plasmid gene deletions on chlamydial replication and/or inclusion morphology ^11– 15^. This simple approach has been fruitful in some respects but also suffers from severe limitations, e.g. unknown polar effects (transcriptional effects of deletions on adjacent genes), unwitting deletion of noncoding small RNAs and off-target changes leading to potential misinterpretation of the properties of individual genes and/or their products. Regardless, *C. trachomatis* and *C. muridarum* ‘mutants’ carrying specific plasmid gene deletions have been used in a large number of such studies of chlamydial pathogenicity, as reviewed by Zhong ^16^.

In previous studies of *C. trachomatis*, we noted that naturally occurring, plasmid-free isolates displayed altered growth cycles, with longer lag phases and lower yields than their partners bearing recombinant shuttle plasmids ^17^. It seems paradoxical that those strains of *C. trachomatis* bearing plasmids have shorter developmental cycles despite carrying a significantly increased metabolic/genetic burden due to the replication of multicopy plasmids and/or larger shuttle vector plasmids. By contrast, all published studies performed with *C. muridarum* indicated that plasmid-free strains had similar growth properties (i.e. inclusion formation and replication kinetics) as the wild-type, plasmid-bearing host ^7, 18, 19^. Whilst characterising the growth characteristics of *C. muridarum,* we noticed that these descriptions of *C. muridarum* are in conflict with our observations. This is, we believe, of critical importance since growth kinetics are a central characteristic of the chlamydial phenotype and both reflect complex intracellular interactions and influence many aspects of experimental design, including studies on pathogenicity. In this study, our aim was to accurately compare and contrast the *C. muridarum* plasmid-free phenotype with that of a chromosomally isogenic plasmid-bearing strain, over time, by recording several parameters. We measured infectivity and chromosomal replication by quantitative PCR under strictly controlled conditions. In parallel to these quantitative studies, we have carefully monitored inclusion formation by confocal microscopy and transmission electron microscopy. By combining these observations, we have advanced the definition of the chlamydial phenotype away from the simple static observation of mature inclusions and have set out to define a plasmid-carrying phenotype based on growth kinetics and on inclusion morphology as it changes with time during the development cycle.

## Methods

### Chlamydia infection and cell culture

There are several distinct strains of *C. muridarum,* which display a range of phenotypic and virulence diversity ^20^. We have selected the strain Nigg Atherton II, hereafter called ‘ *C. muridarum* Nigg P+’ for simplicity of nomenclature, for which there is a defined genome sequence and which was initially plaque purified by Dr. Kyle Ramsey in Chicago. This strain very likely has the same origin as the ‘Nigg’ strains used by others in *C. muridarum* plasmid curing and subsequent genetic experiments although the full passage history is not entirely clear in these publications ^7, 13^.

*C. muridarum* strain Nigg II was provided by Prof. R. Rank to Prof. Kyle Ramsey and originally obtained from Prof. A. Barron ^1^. This isolate was previously known as the causative agent of mouse pneumonitis. It was plaque-purified three times to ensure clonality. The clonal, plasmid-bearing strain is specifically referred herein as *C. muridarum* P+. It was grown in McCoy cells (NCTC, Public Health England, UK) in DMEM supplemented with 10% foetal calf serum containing cycloheximide at 1μg/ml ^12^. EBs were centrifuged onto cells at 754g (Beckman Coulter Allegra X-15R centrifuge) for 30 minutes at room temperature in various formats that included T25 tissue culture flasks, 96 well trays for qPCR and 12 well trays for infectivity assays. The infected cells were then overlaid with culture medium containing cycloheximide (1μg/ml) and gentamicin (20μg/ml) and incubated at 37°C in 5% CO _2_. This strain was cured of its plasmid using novobiocin as described previously ^21^. The resulting plasmid-free isolate *C. muridarum,* designated *C. muridarum* P- was also subject to three rounds of plaque purification and the absence of the plasmid verified by PCR and whole genome sequencing.

Stocks of *C. muridarum* P+ and P- were prepared as described previously ^21^ and titres were determined as described in the growth kinetics and infectivity assay sections. *C. muridarum* and McCoy cells were routinely tested for mycoplasma contamination by fluorescence microscopy and using the Lookout Mycoplasma PCR detection kit (Sigma, UK).

### Transmission electron microscopy

McCoy cell monolayers in 25cm ^2^ culture flasks were infected with plasmid-bearing and plasmid-free strains of *C. muridarum* at an MOI =3.0, this high ratio was only used for the TEM studies to maximise the chances of cutting through the centre of an inclusion with the diamond knife. Cell infection was as described above. At 28 hours post-infection cells were trypsinised, pelleted by centrifugation at 150g for 5 minutes and then resuspended in fixative (4% formaldehyde/3% glutaraldehyde in 0.1M PIPES buffer pH 7.2) for at least 1 hour. Cells were then centrifuged at 7,800g for 5 minutes (Fisher accuSpin Micro 17 centrifuge) into a drop of 5% sodium alginate and then added to an equal volume of 0.1M calcium chloride and left to set for 30 minutes. Embedded cell pellets were then prepared for TEM. Pellets were washed with 0.1M PIPES buffer at pH 7.2 and post-fixed in 1% osmium tetroxide in 0.1M PIPES buffer at pH 7.2 for 1 hour. Pellets were washed again and then *en bloc* stained with 2% aqueous uranyl acetate for 20 minutes before dehydrating in increasing concentrations of ethanol. Acetonitrile was added and incubated for 10 minutes and the pellet then left in 50:50 acetonitrile:Spurr’s resin overnight. Spurr’s resin (100%) was added for 6 hours and then the pellet was embedded in fresh Spurr’s resin, which was allowed to polymerise at 60°C for 24 hours. Thin sections (60 to 90nm gold) were cut and stained on the grid with Reynolds' lead citrate. Negatively stained and thin-section grids were examined in a Hitachi H7000 transmission electron microscope.

### Confocal microscopy

McCoy cells seeded onto glass coverslips in 12 well trays were infected with *C. muridarum* P+ and plasmid-free *C.muridarum* P- (at an MOI=1.0) as described above, and then fixed at 4-hour time intervals between 0 and 40 hours post-infection using 4% paraformaldehyde for 15 minutes. Cells were washed in PBS and permeabilised in saponin buffer (0.1% saponin, 10% foetal calf serum, 0.1% sodium azide) for 1 hour at 4°C. Primary and secondary antibodies were added to the coverslips, and incubated in saponin buffer for 1 hour at room temperature; the coverslips were washed in saponin buffer between steps. A mouse monoclonal primary antibody raised against genus-specific LPS (Chlamydia Biobank Cat. No. #CT601 RRID: AB2721933) was diluted 1:1,000 and combined with an anti-mouse-Alexa Fluor® 488 conjugate secondary antibody (Invitrogen™ Cat. No. A11001 RRID AB_2534069) was used (1:200 dilution) to visualize *C. muridarum*. Cells were counterstained with 1 µg/ml DAPI (Fisher Scientific) and Wheat Germ Agglutinin Alexa Fluor® 594 conjugate (Invitrogen™ Cat. No W11262)), washed a final time in PBS and mounted onto slides with Mowiol 4-88 (Sigma). Images were captured using a Leica TCP SP5 confocal microscope.

### Growth kinetics and infectivity assay using X-gal staining

McCoy cells grown to confluence in 12 well trays were infected with *C. muridarum* P+ and P- at an MOI=1.0 as described above and then harvested at 4 hour time points between 0 and 36 hours post-infection. At each time point, cells were detached from the well through scraping with a sterile 1ml tip, glass beads were added before agitation for 1 min to release the elementary bodies from the cells. The suspension was added to an equal volume of 4 x Sucrose Phosphate (4SP) and stored at -70°C. To assess infectivity at each time point, *C. muridarum* was titrated in 10-fold dilutions on a 96 well tray and fixed at 28 hours post-infection using 100% methanol for 20 minutes at -20°C. Full details of the infectivity assay are described in Skilton *et al.* ^22^. Briefly, a mouse monoclonal primary antibody raised against genus-specific LPS (Mab29) was incubated with the infected cells overnight at 4°C. The cells were then washed with PBS and incubated with an anti-mouse antibody conjugated with β-galactosidase (Calbiochem) for 1 hour at 37°C. For staining, 100μl of a staining solution [5.0mM K3Fe(CN)6, 5.0mM K4Fe(CN)6·3H2O, 2.0mM MgCl2·6H2O, 0.25M 5-bromo-4-chloro-3-indolyl-β-d-galactopyranoside (X-Gal)] was added per well and incubated for 4 hours at 37°C. The chromogenic X-Gal substrate generated blue-stained *C. muridarum* inclusions, which were then counted and titres were calculated.

### Time course of infection for qPCR analysis

McCoy cells grown to confluence in 96 well trays were infected with *C. muridarum* P+ and P- at MOI = 1.0 as described above. For each time point, cells were infected in quadruplicate, and the infection was stopped at 0, 4, 8, 12, 16, 20, 24, 28, 32, and 36 hours post-infection. At each time point, a tray was stored at -70°C for subsequent nucleic acid extraction.

### Chromosome quantification by real-time quantitative PCR

The quantification of chromosomal DNA at each time point was accurately determined by performing 5′-exonuclease (TaqMan) assays with unlabelled primers and carboxyfluorescein/carboxytetramethylrhodamine (FAM/TAMRA) dual-labelled probes. A pan chlamydial PCR assay was developed, primers and probe sequences were as follows,: **CM_omcB_F** (5’-GGAGATCCTATGAACAAACTCATC-3’), **CM_omcB_R** (5’-TTTCGCTTTGGTGTCAGCTA-3’), **CM_omcB_Probe** (5’-FAM-CGCCACACTAGTCACCGCGAA-TAMRA-3’).Five microliters of each sample was added to 20µl reaction mixture containing forward primer (400nM), reverse primer (400nM), probe (200nM) and SensiFAST Probe Lo-ROX mix (Bioline). Real-time PCR cycles (95°C for 5 minutes, followed by 40 cycles of 95°C for 10 seconds and 60°C for 50 seconds) were performed in a 7500 Fast Real-Time PCR System (Applied Biosystems).

A standard curve was prepared using plasmid DNA prepared in *E.coli* (pSRP1A) containing the *omcB* gene from *C. trachomatis* L1, which has identical priming sites in the *C. muridarum* chromosome ^23^.

### Chromosome and plasmid sequencing

Sequencing of the *C. muridarum* P- chromosome was performed using Illumina MiSeq at the Sanger Centre, Cambridge, UK, with multiplexing using paired read lengths of between 75 bp and 100 bp, giving a depth of coverage of 5x for the plasmid-free genome. The raw sequence data can be accessed from the European Nucleotide Archive, accession number ERS351386. *De novo* genome assembly of the resulting reads was performed using Velvet (version 1.2.09) for short paired reads, with a chosen K-mer length of 73. The final results gave 23 nodes and an N50 of 1,051,043. The assembly was ordered against reference genome NC_002620 (which includes the plasmid sequence) using ABACAS (version 1.3.2)(Algorithm Based Automatic Contiguation of Assembled Sequences; SourceForge). No plasmid sequence reads were found. The annotation of reference sequence NC_002620 was then transferred to the *C. muridarum* P- assembly. Genes TC_0412 and TC_0236 were identified and checked for the SNPs previously suggested as being involved in restoration of infectivity in plasmid-free isolates ^24, 25^.

Plasmid pGFP::Nigg was constructed from pSW2NiggCDS2 ^21^, which has a unique *Spe* I site within CDS2 (from pNigg) and a unique *Mlu* I site in CDS 1 (from pSW2). The *Spe*I- *Mlu*I fragment in pSW2NiggCDS2 (including CDSs 3-8 from pSW2) was replaced with the *Spe*I- *Mlu*I fragment by PCR using primers CDS2_F( *Spe*I): 5’- TCC AGA ACT AGT TAC GAA GAC CAA AC -3’ and CDS1_R( *Mlu* I): 5’-aaaaaa acgcg T CTCCAAAAGTTAGGAATAGCCTACTTCT -3’, and total genomic DNA of *C. muridarum* Nigg P+(containg pNigg) as the template. The resulting plasmid contained the entire pNigg CDS2-8 and also 48nt of pNigg CDS1 (from the start codon) which was in-frame and fused with truncated pSW CDS1 at the *Mlu*1 site. Plasmid pGFP::Nigg DNA was sequenced verified using the complete plasmid sequencing service using Next-Generation sequencing technology at Massachusetts General Hospital CCIB DNA Core, Cambridge MA, USA. The complete sequence of pGFP::Nigg is available as a FastA file ( Supplementary File 1).

### Statistical analysis

One step growth curves and chromosomal replication graphs were produced in GraphPad Prism (GraphPad Software, USA, version 7.0.3.0), inclusion size measurement from confocal images was executed in ImageJ (NIH, USA, version 1.8.0_112) and all remaining statistical calculations were performed in Excel (Microsoft, USA, version 15.0.4989.1000). All values are expressed as means ± standard deviation (SD) of two to four replicates. Comparisons of means were performed by using a two-tailed Student *t-*test for independent samples. The single or multiple inference significance level was set to 5%.

## Results and discussion

### Plasmid curing and genomic sequence of the resultant *C. muridarum* P- strain

The plasmid-bearing wild-type *C. muridarum* P+ strain was cured of its endogenous plasmid using novobiocin ^21^. To ensure purity and experimental rigour, this plasmid-cured isolate was plaque purified three times (using cycloheximide in the media) and designated *C. muridarum* P-. During this process, we did not observe significant differences in the sizes of plaques produced by plasmid-cured clones of *C. muridarum* P- in McCoy cells compared to the wild-type isolate. Both formed plaques of similar size; plaque characteristics of both the parental strain *C. muridarum* P+ and its plasmid-free derivative strain, *C. muridarum* P-, at six days post infection are shown in Figure S1.

Supplementary file 2

Comparison of plaque assay protocols has revealed differences in plaque sizes and morphology, dependent on the method used ^26^. All experiments described in this study were performed using a standard protocol using cylcoheximide in the culture medium. It is noteworthy that whilst *C. muridarum* can efficiently infect cells without the need for centrifugation, we have incorporated this step to ensure reproducibility of conditions. The use of cycloheximide removes variable and uncontrollable inter-experimental host effects (e.g. cell division) by blocking host translation. Plaquing using our simple, standard agarose overlay protocol takes place over extended periods of time and up to six days. Plasmid-cured *C. muridarum* have been described with either small plaque phenotypes ^7, 19^ or normal plaques ^18, 25^. Many subtle variations are reported in the plaque assay protocols, including choice of cells, incubation times and infection process, all of which may account for the differences observed in plaque sizes and reported by others in *C. muridarum*. In fact the use of cycloheximide has been reported to affect chlamydia cell lysis processes ^27^ and this may be a factor explaining why we only found *C. muridarum* with normal plaque phenotypes.

We observed that plaque size was dependent on incubation time and plaque morphology was also variable. Whilst plaques appeared circular to the naked eye, higher magnification revealed variations ( Figure S1B) and thus plaque diameter was not a constant nor a consistent characteristic. Thus plaque assays were used only for ensuring clonality of the strains used in this study. Furthermore, since plaque formation was variable and plaques often overlapped, our evaluations of infectivity in this study were determined through manual count of inclusions stained with a chlamydial genus-specific monoclonal antibody (inclusion forming units). Furthermore, a plasmid deficient *C. muridarum* has been shown to have reduced infectivity/ plaquing efficiency but no difference in its ability to form inclusions ^18, 27^. Measuring inclusion forming units has proven an enduring, reproducible and accurate way of defining the infectivity of viable preparations of chlamydia ^28, 29^.

*De novo* assembly of the *C. muridarum* P- genome sequences and ordering of these against the reference genome NC_002620 showed that the *C. muridarum* P- sequence was free of any plasmid reads as expected, and there were no chromosomal rearrangements in comparison to the reference sequence. Mutations identified in CM3.1 and CM972 ^24^ have been linked to restoration of infectivity in plasmid-free isolates. In TC_0412 there was a single nucleotide insertion (T) in *C. muridarum* P-, 14bp upstream of that found in CM3.1 and CM972, causing a frame shift mutation. However, this same mutation was identified in the wildtype parent strain chromosome (GCA_000174975.1), precluding it from any involvement in the restoration of infectivity in plasmid-free strains. The mutation identified in TC_0236 in CM3.1 ^25^ was absent in *C. muridarum* P-.

### The morphological phenotype of inclusions formed by *C. muridarum* P- defined by phase contrast and electron microscopy

To pinpoint the formation of the bull’s eye phenomenon, images of developing inclusions were taken across a time course by phase contrast microscopy to discover when the bull’s eye phenotype was most obvious in *C. muridarum* P- infected McCoy cells. The bull’s eye phenotype becomes clearly apparent (~90% inclusions) at 28 hrs post infection while the equivalent inclusion morphology of the cognate plasmid bearing strain at this time point shows mainly wild – type inclusions.

**Figure 1.**
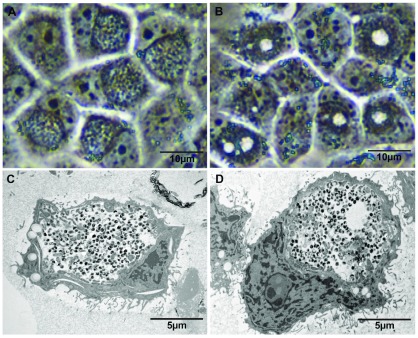
Phase and electron micrograph images of *C. muridarum* inclusions. Phase contrast microscope ( **A** & **B**) and transmission electron microscope ( **C** & **D**) images of *C. muridarum* P+ ( **A** & **C**) and P- ( **B** & **D**) inclusions at 28 hours post infection in McCoy cells. ‘Bulls-eye’ phenotype of the plasmid-free strain can be seen in panels **B** & **D**.

Mature inclusions of plasmid-cured *C. muridarum* P- at 28hrs post infection displaying the bull’s eye phenotype characteristic of plasmid-free isolates of *C. muridarum* P- are shown in Figure 1. By contrast *C. muridarum* P+ displayed the standard, well-recognised regular mature wild-type inclusion morphology characteristic of this host. The time course analyses were stopped at 36 hrs post infection when the developmental cycle and DNA replication were complete, at this time there were no discernible differences in host cell lysis between P+ and P- as measured by phase contrast microscopic examination.

The phenotype of normal inclusions of plasmid-bearing *Chlamydia* have been well characterised ^3^. However, detailed description of the phenotype of the bull’s eye inclusion from naturally occurring plasmid-free or plasmid-cured isolates has proven elusive and hence difficult to define. There is only one description of a plasmid-free inclusion from *C. trachomatis* by transmission EM ^30^. This description is from a naturally occurring plasmid-free strain and no TEM image of inclusions from plasmid-cured isolates have been reported in the literature. EM is useful as it allows for the level of resolution required to observe individual EBs and RBs. We noted that the bull’s eye phenomenon was a transient effect as it was not evident early in infection nor was it visible in every inclusion; presumably the orientation and size of the inclusions with respect to the light path may explain this phenomenon.

To look deeper into the inclusion structure of the plasmid-free phenotype in *C. muridarum* we used TEM to achieve higher resolution of the inclusion. To maximise the chances of slicing through an inclusion, we used a higher MOI (MOI=3.0), and to preserve inclusion structure, the infected cells were mixed with alginate prior to fixing and embedding in EM resin.

Figure 1 shows the morphology of mature inclusions from *C. muridarum* P- and its wild type counterpart *C. muridarum* P+ examined by phase contrast microscopy and EM. Profound differences are visible between inclusions formed by plasmid–cured and wild type *C. muridarum* in terms of basic morphology as revealed by these analyses.

### Confocal analysis of the developmental cycle of *C. muridarum* P-

Our initial studies aimed at describing the morphology of inclusions formed by *C. muridarum* P- showed that there were differences in the rate of development of inclusions between the P + and P- strains. These observations stand in contradiction with previous kinetic studies using plasmid-cured *C. muridarum,* which reported no difference between plasmid bearing and plasmid-cured strains ^7, 18, 19^.

**Figure 2.**
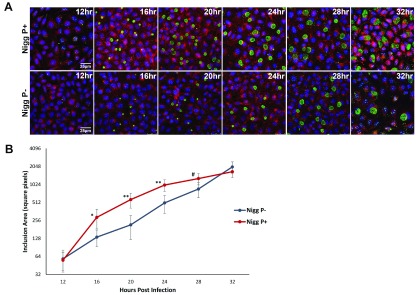
*C. muridarum* developmental cycle visualised by confocal microscopy. Confocal microscope images ( **A**) of *C. muridarum* P+ and P- infected McCoy cells at set time intervals in the developmental cycle and quantification of the inclusion sizes seen ( **B**). Mouse monoclonal primary antibody raised against genus-specific LPS (Mab29) detected with anti-mouse Alexa Fluor® 488 conjugate secondary antibody staining inclusions green. Cells were counter-stained using wheat germ agglutinin Alexa Fluor® 594 staining the plasma membrane (red) and DAPI to identify the cell nucleus (blue). Mean and standard deviation of inclusion size, expressed as square pixels, measured from the confocal microscope pictures during the course of 1-step growth curve using ImageJ software. * *p*<0.01, ** *p*<0.00001, # *p*<0.0001.

Whilst EM is useful to define structures, the extensive fixation processes and the limited chances of cutting thin sections through the centre of inclusions make this approach to study the morphology of inclusion development difficult. Thus to plot precisely the changes in inclusion size within a dynamic framework and at higher resolution than phase contrast, we investigated the process of inclusion formation by confocal microscopy during the developmental cycle. For consistency and reproducibility, we used the same reagents in this analysis as in later infectivity assays. Thus we used the monoclonal antibody Mab 29, which is directed at chlamydial LPS. Here the fluorescence signal recorded during imaging of confocal microscopy masked the bull’s eye appearance of the inclusions. Time courses of infection for *C. muridarum* P+ and *C. muridarum* P- by confocal microscopy of inclusions are shown in Figure 2a and 2b, respectively. Inclusion area were measured using the open platform scientific analysis software Image J version 1.8.0_112.

Our images showed conclusively that inclusion size in P- is significantly smaller than in P+ at the same time points up to 28hrs post infection in the developmental cycle. This demonstrates that the phenotype of a plasmid-free strains should be considered not just at a single time point at or near the end of the developmental cycle, but as a series of frames encompassing differences in inclusion size and morphology throughout the time course of infection. Consistent with overall larger inclusion size at 32 hrs post infection, the maturation of inclusions for *C. muridarum* P- occurs 4hrs later than for *C. muridarum* P+. Whilst detailed morphological time courses of inclusion development have not been previously performed on *C. muridarum* P- , the data confirmed that there were clear differences in the overall growth profile of P- and P+, with *C. muridarum* P- initially showing smaller inclusions and delayed inclusion development. These subtle but critical differences are in stark contrast to analyses from previous studies, which concluded that the developmental cycle is identical in duration and yield for *C. muridarum* plasmid-bearing and plasmid-cured clones ^7, 18, 19^.

### One step growth kinetics

Bacterial fitness reflects the ability of a microorganism to adjust its metabolism to suit growth conditions. Measuring growth rates under defined culture conditions is an objective way of assessing fitness and deduced generation time is the optimal measure to compare the fitness of strains. In our experience, both chlamydial plaque size and plaque morphology are unreliable parameters for characterising differences between chlamydial strains. Chlamydial plaque assays of the same strains are variable and dependent on perfectly optimised conditions to obtain accurate enumeration of infectivity ^26^. The results also depend on the physiological quality of the cells. For consistency we always use cycloheximide when culturing plasmid-free isolates, thus our preferred approach to obtain accurate chlamydial infectivity titres was to use an assay using objective quantifiable factors, which removes potential observer bias from the measurements. Therefore to perform accurate growth kinetic analyses, infectivity was measured by inclusion staining assay. These assays were performed in quadruplicate so that statistically significant data could be obtained allowing accurate measures of infectivity. This technique avoids the need for plaque formation, only detecting a primary infection and is thus highly sensitive and provides accurate quantifiable data. Since LPS is essential for chlamydia survival, all inclusions were stained, regardless of size and were clearly identifiable by their discrete and distinctive staining profile/morphology within the cell cytoplasm ^22^.

One step infectivity growth curves were performed in 24-well trays using carefully titred inocula of P+ and P-. To ensure species compatibility, murine McCoy cells were used and infected at an MOI of 1. Samples were taken for infectivity assay at four hourly periods from 8hrs post infection to the end of the experiment.

**Figure 3.**
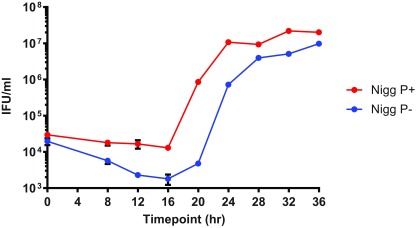
Comparison of infectivity growth profiles for *C. muridarum* P+ and P- strains. One step growth curves measured by inclusion assay of infectious EBs. Infected McCoy cells were removed for analysis at 4 hourly time intervals and then titrated to assess the quantity of inclusion forming units. Inclusions were stained and counted using a mouse monoclonal primary antibody raised against genus-specific LPS (Mab29) detected with anti-mouse β-galactosidase conjugate secondary antibody staining inclusions blue in the presence of X-gal. All values are expressed as means ± standard deviation (SD) of two replicates.

Consistent with the confocal microscopical observations, which showed smaller inclusions of the *C. muridarum* P- in all the early stages (up to 28hrs) of the developmental cycle, the growth kinetics showed subtle differences in one step infectivity growth curves between P+ and *C. muridarum* P- as seen in Figure 3. *C. muridarum* P- had a 4 hr longer eclipse period and whilst its doubling time was not significantly different, the yield of infectious progeny was five times lower than its plasmid-bearing cognate counterpart P+. This observation is in contrast to the reported results comparing developmental cycles of plasmid cured *C. muridarum* in other laboratories using plaque assays ^7, 18, 19^.

Re-examination of the published growth kinetics data for plasmid-cured ‘small-plaquing’ strain *C. muridarum* CM972 and its larger plaquing derivative CM3.1 indicated that there are in fact growth profile differences; despite the original claim by the authors ^18^. Even though this study was performed using the lower efficiency plaque assay procedure, figures clearly showed that plasmid-cured isolates have a 25% longer eclipse phase relative to the plasmid bearing parent, thus we believe the conclusion that there are no differences between plasmid cured and plasmid bearing isolates is incorrect ^18^. The data from the other studies where no differences between P + and P- growth profiles are reported did not provide data of sufficient resolution to allow a re-evaluation ^19^.

**Figure 4.**
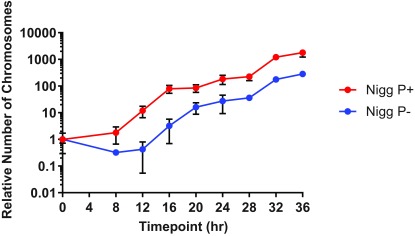
Chromosomal replication during the developmental cycle of *C. muridarum* P+ and P-. Infected McCoy cells were removed for qPCR analysis at 4 hourly time intervals and the relative number of chlamydial genomes were determined using the *C. muridarum* omcB assay. All values are expressed as means ± standard deviation (SD) of four replicates.

Chromosomal replication was measured by qPCR analysis, which gives an accurate numerical measure of the replication of the DNA, providing an additional quantifiable objective and precise measure of the developmental cycle. Because replication occurs before infectious EBs are formed, the gradients of the qPCR curves were not as pronounced as observed with the infectivity curves, which measured infectious EBs through IFU. Nevertheless the qPCR analysis verified both the longer ‘eclipse’ period and the reduced yield for *C. muridarum* P- as shown in Figure 4.

The doubling time of *C. muridarum* P+ and P-, defined as *t*/χ where *t* is the hours of exponential growth and χ is the number of generations, was calculated by analysing the 1-step growth curves for infectivity (IFU) as well as chromosome replication. IFU measurement showed that during the exponential growth phase, (when RBs are dividing and differentiating into infectious EBs), *C. muridarum* P+ and P- had an estimated doubling time of 47 and 83 minutes respectively, (16 to 24 h.p.i. for P+ and 20 to 32 h.p.i. P-), suggesting a longer generation time (although this was not statistically significant) for the plasmid-cured *C. muridarum* (see Table S1). By contrast, differences were much less noticeable when the entire development cycle was taken into account (182 and 205 min for P+ and P-, respectively), falsely implying a similar overall growth behaviour in both cases.

Conversely, measurements of *C. muridarum* P+ and P- chromosome replication provided no evidence of a longer doubling time for P- during either the exponential phase (84 and 87 min for P+ and P-, respectively) or the whole development cycle (296 and 237 min for P+ and P-, respectively). This last observation can be explained by the fact that DNA replication begins at an earlier stage than both RB division and differentiation into EBs; hence, this phenomenon smoothens the typical sigmoidal ‘infectivity’ growth curve.

### Tranformation of *C. muridarum* P- with pGFP::Nigg

**Figure 5.**
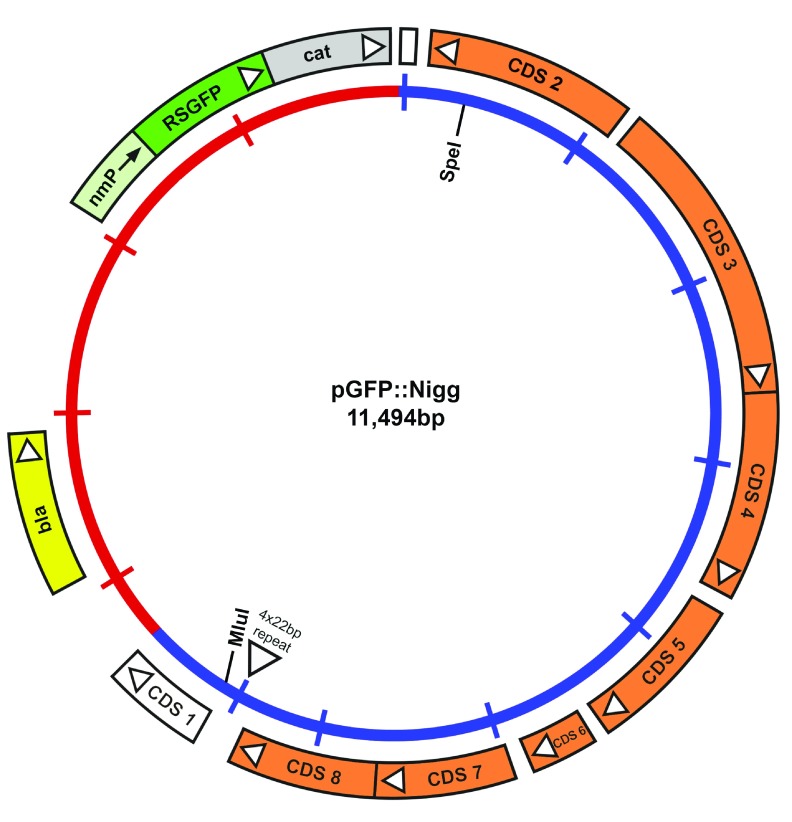
Map of the plasmid vector pGFP::Nigg. The inner circle represents the chlamydial plasmid (blue) and the vector sequences (red). The coding sequences and their direction of transcription are represented by the boxes of the outer circle. The CDS2-8 were from pNigg in *C. muridarum* Nigg. The *cat* gene is fused with RSGFP (green) and expressed by a promotor derived from *Neisseria meningitides* (nmP).

To prove that the loss of the plasmid was the sole cause of the change in growth and chromosomal replication profile, it was necessary to re-introduce the plasmid into the *C. muridarum* P-. Therefore we constructed a recombinant plasmid pGFP::Nigg using a previously described strategy for making recombinant shuttle plasmids in *C. trachomatis* ^17^. The map of the plasmid is shown in Figure 5.

The resultant recombinant plasmid was verified by sequence analysis, the nucleotide sequence of pGFP::Nigg is presented in Supplementary File 1.

Plasmid pGFP::Nigg is a vector constructed specifically for *C. muridarum* transformation. Plasmid pGFP::Nigg is similar to the widely used *C. trachomatis* vector pGFP::SW2; the only difference is that the pSW2 CDSs 2-8 in pGFP::SW2 have been replaced with pNigg CDSs 2-8.

The construction of pGFP::Nigg was based on plasmid pSW2NiggCDS2, which was recovered from *C. muridarum* P+ (‘wild-type’ strain carrying pNigg) transformed with plasmid pGFP::SW2. The recovered plasmid pSW2NiggCDS2 contains only CDS2 from pNigg which has replaced the pSW2 CDS2 in pGFP::SW2 ^21^.

Since CDS1 is essentially redundant in *C. trachomatis* ^31^ (and other chlamydial species e.g. *C. pneumoniae* ^32^) we expected that insertion of the *E.coli* shuttle vector within this gene would have little or no effect on the properties of the plasmid, as demonstrated with similar recombinant *C. muridarum* shuttle plasmids constructed by others pBRCM ^33^ and pGFP::CM ^13^ which were also cloned via CDS1. *C. muridarum* P- transformed by pGFP::Nigg was selected with penicillin and resulted in the development of green inclusions; it was additionally chloramphenicol resistant. Transformation of *C. muridarum* P- with pGFP::Nigg restored the wild type inclusion phenotype when grown in McCoy cells without antibiotics.

**Figure 6.**
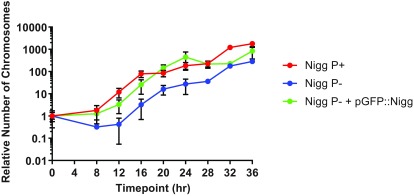
Graph showing the effect of restoring the plasmid to *C. muridarum* P- on chromosomal replication. *C. muridarum* Nigg P- was transformed with pGFP::Nigg and used to infect McCoy cells. Samples were removed for qPCR analysis at 4 hourly time intervals and the relative number of chlamydial genomes were determined using the *C. muridarum* omcB assay throughout the developmental cycle.

A one step DNA replication assay of *C. muridarum* P- transformed by pGFP::Nigg was performed and compared with chromosomal replication one step curves for *C. muridarum* P- and P+ as shown in Figure 6.

Analysis of chromosomal DNA replication of *C. muridarum* P- transformed by pGFP::Nigg showed that the recombinant plasmid restored the shorter eclipse period and the replication profile from 8 to 20hrs post infection was similar to the *C. muridarum* P+, but then chromosomal replication flattened.

These data indicate that the plasmid plays a key role in determining the overall growth profile because a shorter eclipse phase of the growth kinetics is restored.

## Conclusions

1. The discovery and definition of subtle phenotypic differences between plasmid bearing and plasmid cured isolates is important as it could have critical impact on chlamydial growth, affecting experimental design, *in vivo* research and vaccine development. These differences could be species or even strain specific, thus caution should be exercised in making generalisations about specific plasmid phenotypes.
2. We have tried to replicate work from previous literature on the study of *C. muridarum* and been unable to reproduce specific outcomes regarding inclusion development and growth kinetics. Reproducibility is a fundamental scientific principle, thus all our experimental data is made available here for others to repeat the findings, together with our full protocols, which we hope will enable open debate of these critical issues and guide the field in the future. Reproducible methods and high quality data are essential to ensure that research findings are based on the best possible evidence and we conclude that accurate and highly reproducible methods should be included in future studies on plasmid phenotypes.
3. It is now timely and appropriate to advance the definition of the *Chlamydia* plasmid-related phenotypes away from the simple notion of the static observation of mature inclusions or purified EBs at a single time point. It is necessary to re-define plasmid-based phenotypes by careful and accurate analysis and comparison of growth kinetics and, where necessary, combine these with morphological observations of inclusion structure. The sampling of multiple, regular time points covering the whole developmental cycle for infectivity, DNA replication and gene expression is necessary. The use of single data points and even the selective use 3 or 4 time points for sampling is insufficient. A rigorous multiple time point sampling approach is necessary for investigating specific protein expression/regulation. We believe that this level of experimental rigour should be the standard as part of accurate new protocols for measuring phenotypic characteristics of chlamydial mutants.
4. From applying these principles we conclude that the pNigg plasmid alone is responsible for the differences observed in growth properties observed between *C. muridarum* P+ and P-.

## Data availability

Data are available at OSF: DOI 10.17605/OSF.IO/74E2Z | ARK c7605/osf.io/74e2z

The *C. muridarum* P- ** chromosome raw sequence data can be accessed from the European Nucleotide Archive, accession number ERS351386.

## Supplementary material

**Supplementary File 1. Complete sequence of the plasmid vector pGFP::Nigg.**

**Figure S1. Plaques of *C. muridarum* P+ and P-.** McCoy cells infected with *C. muridarum* P+ ( **A**) and *C. muridarum* P- ( **B**) at the same multiplicity of infection were stained with neutral red at 6 days post infection. The assay was performed in 6-well tissue culture trays and individual plaques are indicated by arrows. Higher magnification (4X) of neutral red stained plaques are shown. The figure is representative of 3 images (see data).

**Table S1. Doubling time of *C. muridarum* NiggP+, NiggP- and NiggP- +pGFP::Nigg during either the exponential phase or the whole development cycle.** ( **A**) RB replication; ( **B**) Genome replication. All values are expressed as means ± standard deviation (SD) of two/four replicates. Comparison of means was performed by using a two-tailed Student’s t-test for independent samples.
