# Supplementary figures and images for: The Chlamydia muridarum plasmid revisited : new insights into growth kinetics
Source: Wellcome Open Res. 2018 Mar 8;3:25. [Version 1] doi: 10.12688/wellcomeopenres.13905.1 (PMC5871946; doi:10.12688/wellcomeopenres.13905.1)

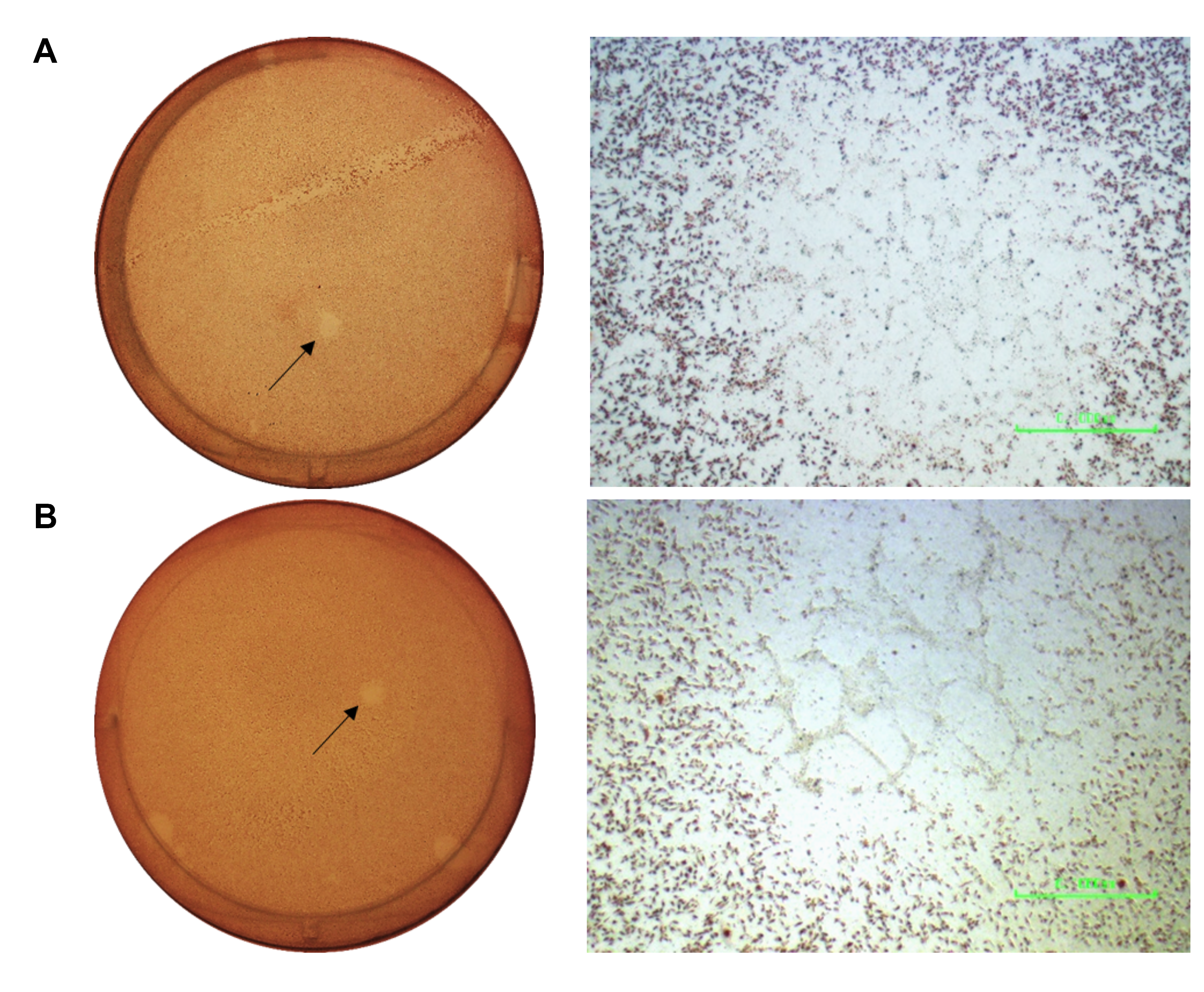

Supplement: Supplementary file 1 [file wellcomeopenres-3-15117-s0001.tgz › fb95314e-3ff1-47a3-afa0-6822d9a3b3e4.tif]
