# Supplementary material for: The Chlamydia muridarum plasmid revisited : new insights into growth kinetics
Source: Wellcome Open Res. 2018 Mar 8;3:25. [Version 1] doi: 10.12688/wellcomeopenres.13905.1 (PMC5871946; doi:10.12688/wellcomeopenres.13905.1)
Supplement: Supplementary file 2 [file wellcomeopenres-3-15117-s0002.tgz › 37a31bc8-4e55-45c2-9cec-575b80fdebb2.docx]

| **A)** | **Exponential Phase** | | | | | | |
| --- | --- | --- | --- | --- | --- | --- | --- |
|  | **Number of Inclusions (IFU/mL)** | | **Time (min)** | **Number of Generations (χ)** | | **Doubling Time (min)** | |
|  | **t_0_** | **t_1_** |  |  |  |  |  |
| **NiggP+** | 4.19x10^3^±0.56x10^3^ | 4.82x10^6^±0.52x10^6^ | 480 | 10.2 | | 47.2 | |
| **NiggP-** | 7.69x10^3^±1.31x10^3^ | 3.19x10^6^±0.86x10^6^ | 720 | 8.7 | | 82.7 | |
|  |  |  |  |  |  |  |  |
|  | **Whole Development Cycle** | | | | | | |
|  | **Number of Inclusions (IFU/mL)** | | **Time (min)** | **Number of Generations (χ)** | | **Doubling Time (min)** | |
|  | **t_0_** | **t_1_** |  |  |  |  |  |
| **NiggP+** | 2.69x10^3^±0.56x10^3^ | 9.88x10^6^±0.25x10^6^ | 2160 | 11.9 | | 181.5 | |
| **NiggP-** | 2.31x10^3^±0.56x10^3^ | 3.30x10^6^±0.25x10^6^ | 2160 | 10.5 | | 205.7 | |
|  | t_0_, initial inclusion number; t_1_, final inclusion number | | | | | | |
|  |  |  |  |  |  |  |  |
|  |  |  |  |  |  |  |  |
| **B)** | **Exponential Phase** | | | | | | |
|  | **Genomic copy number/mL** | | **Time (min)** | **Number of Generations (χ)** | | **Doubling Time (min)** | |
|  | **t_0_** | **t_1_** |  |  |  |  |  |
| **NiggP+** | 2.16x10^2^±1.11x10^2^ | 9.55x10^3^±2.55x10^3^ | 480 | 5.7 | | 84.2 | |
| **NiggP-** | 1.87x10^2^±1.41x10^2^ | 7.11x10^3^±2.82x10^3^ | 480 | 5.5 | | 87.2 | |
| **NiggP- +pGFP::Nigg** | 1.30x10^3^±0.69x10^3^ | 1.75x10^5^±1.08x10^5^ | 720 | 6.9 | | 104.34 | |
|  |  |  |  |  |  |  |  |
|  | **Whole Development Cycle** | | | | | | |
|  | **Genomic copy number/mL** | | **Time (min)** | **Number of Generations (χ)** | | **Doubling Time (min)** | |
|  | **t_0_** | **t_1_** |  |  |  |  |  |
| **NiggP+** | 1.44x10^3^±0.57x10^3^ | 2.14x10^5^±0.57x10^5^ | 2160 | 7.3 | | 295.9 | |
| **NiggP-** | 2.31x10^2^±1.36x10^2^ | 1.25x10^5^±0.19x10^5^ | 2160 | 9.1 | | 237.4 | |
| **NiggP- + pGFP:: Nigg** | 1.30x10^3^±0.69x10^3^ | 3.33x10^5^±1.63x10^6^ | 2160 | 8 | | 270 | |
|  | t_0_, initial copy number; t_1_, final copy number | | | | | | |

**Table S1.** Doubling time of *C. muridarum* NiggP+, NiggP- and NiggP- +pGFP::Nigg during either the exponential phase or the whole development cycle. (**A**) RB replication; (**B**) Genome replication. All values are expressed as means ± standard deviation (SD) of two/four replicates. Comparison of means was performed by using a two-tailed Student’s t-test for independent samples.
