# Supplementary material for: The Chlamydia muridarum plasmid revisited : new insights into growth kinetics
Source: Wellcome Open Res. 2018 Mar 8;3:25. [Version 1] doi: 10.12688/wellcomeopenres.13905.1 (PMC5871946; doi:10.12688/wellcomeopenres.13905.1)
Supplement: Peer review history

# Peer review: Referee response for version 1

**DOI:** 10.21956/wellcomeopenres.15117.r31736

**Published:** 2018-03-26

**Authors:** Derek J. Fisher

**Affiliations:** 1 Department of Microbiology, Southern Illinois University, Carbondale, IL, USA

The manuscript by Skilton *et al*. reports the novel finding that a plasmid-negative mutant strain of *Chlamydia muridarum* is impaired for growth compared to the plasmid-positive parental strain in a cell culture infection model. These results are in contradiction to previous studies with *C. muridarum* plasmid mutants, but are consistent with reports for *Chlamydia trachomatis* plasmid mutants. Additionally, normal growth appears to have been restored through complementation with a newly developed *C. muridarum* shuttle vector. Growth was measured throughout the development cycle via IFU production, inclusion size, and chromosome equivalents. The authors conclude that: 1) plasmid deficient *C. muridarum* is growth impaired, 2) differences in growth can be attributed solely to the presence or absence of the plasmid, 3) growth should be assessed throughout development rather than using end-point analysis or plaque assay, and 4) that assessment of growth at multiple time points throughout development is essential for characterizing mutants and should become standard practice for the chlamydial field for which genetics has only recently become widely applicable.

 This is a well-written and timely manuscript. With the increased use of plasmid-based genetic tools in *Chlamydia* it is important that the field adopts reproducible and rigorous methods for analyzing mutants and this seems to be a clear goal of the authors based on their concluding statements. In addition, the findings from this manuscript, which contradict prior studies, are significant in that the *C. muridarum*-mouse infection model is widely used in the community for exploring chlamydial pathogenesis and an increasing number of studies with mutants are likely on the horizon.

 There are a few technical weaknesses that impact what appears to be a broader manuscript theme – carefully discerning phenotypic differences between mutants through standard methods of analyses – that if addressed would strengthen the manuscript. The proposed replications of specific experiments might also further buttress the argument that growth differences in cell culture do exist between plasmid-positive and plasmid-negative *C. muridarum* matched-strains, a finding that differs from prior studies.

 Major Criticisms:

 The ability to discern phenotypic differences between a panel of strains, in this case the P+ (parental, wild-type), P- (plasmid-cured mutant), and P+pGFP (complemented) strains, would seem to hinge on the use of multiple experimental approaches performed with an appropriate number of biological replicates. As the authors are presenting data that conflicts with prior reports/interpretations and are seeking to lay the foundation for how mutants are to be analyzed in the future, this topic seems particularly pertinent to the experiments reported in this manuscript.

 1) In figure 1, the “Bulls-eye” inclusion phenotype for the P- mutant is clearly shown in both phase-contrast and EM images. The authors report that plasmid-complementation of the P- mutant restores the wild-type inclusion phenotype. Addition of a phase-contrast image of a field of cells infected with the P+pGFP strain should be included with figure 1. This may require a minor restructuring of the manuscript to include the complementation and vector information earlier in the results section. However, this rearrangement is also suggested for addressing the remaining major criticisms.

 2) The inclusion size data shown in figure 2, representing one of the three quantitative approaches used to support differing phenotypes amongst the strains (along with IFU production and genome equivalents), is an essential part of the results. The data show a significant difference in inclusion size at early time points and document a growth defect that would be missed if assessed only at the end stage. However, based on the data provided through the OSF server, the data seem to be derived from a single biological replicate, the 12-hour time point data is derived from only 2 (P+) and 3 (P-) inclusions, and the average number of inclusions measured per time point (excluding the 12 hr) is only 14. The 12-hour time point data might have been limited due to the small size of the inclusions. However, the overall inclusion counts are low, and it is suggested that an additional biological replicate is performed to increase inclusion counts and ensure reproducibility. In addition, inclusion of the P+pGFP strain in the data set would strengthen the claim that all phenotypic differences are due to the plasmid by providing an extra quantitative measurement of the fitness of the complemented strain.

 3) In figure 3, it is not clear why the 32-hour time point was selected for calculating the growth rate of the P- mutant (“20 to 32 h.p.i.”). The 28-hr time point would seem to better reflect the time at which the strain exits from the exponential growth phase.

 4) Figure 3 - Infectivity growth curve data and growth rate calculations should be included for the complemented strain.

 5) It is recommended to merge figures 4 and 6 reporting the genome equivalents for the P+/P- strains (figure 4) and P+/P-/P+pGFP (figure 6) into one figure. The gDNA data for these strains also appears to come from a single experiment that did include four technical replicates. As establishment of reproducible and robust methods for phenotyping mutants appears to be a major focus of the manuscript, a second repetition seems appropriate.

 Minor Criticisms:

 1) The authors state using anti-LPS antibodies. *Chlamydia* technically have lipooligosaccharides (LOS) (PMID 21628561 and 10358025). 

 2) Server data – The Infectivity data table lists a 22 hr time point, which is likely the 20 hr time point data.

 3) While the time points used to determine the growth rate via genome equivalents can be figured out using the server data, it would be easier for the reader if they were listed in the text. P+ 8 to 16, P- 12 to 20, and P+pGFP 12 to 24

 4) The authors present several valid criticisms of the plaque assay when used as a measurement of strain phenotypes. However, I would argue that the plaque assay is still a useful method for assessing the growth phenotype of strains and mutants under a condition that measures growth and infectivity over multiple developmental cycles. This is reflective of what would happen in an animal, albeit in a much more permissive environment. I do think it is crucial that the parameters used for the plaque assay are explicitly stated and critical that, when feasible, matching strain sets (parent, mutant, complement) are used for plaque assay phenotyping in combination with alternative approaches such as those used in this manuscript.

I have read this submission. I believe that I have an appropriate level of expertise to confirm that it is of an acceptable scientific standard, however I have significant reservations, as outlined above.

# Peer review: Referee response for version 1

**DOI:** 10.21956/wellcomeopenres.15117.r31735

**Published:** 2018-03-23

**Authors:** Ming Tan, Julie Brothwell, Derifa Kadouche, Syed Rizvi, Christopher Rosario, Qiang Zhang

**Affiliations:** 1 Departments of Microbiology & Molecular Genetics, and Medicine, University of California, Irvine, Irvine, CA, USA; 2 Department of Microbiology & Molecular Genetics, University of California, Irvine, Irvine, CA, USA

This article nicely analysed growth defects in the intracellular *Chlamydia* infection due to absence of the chlamydial plasmid. Skilton and colleagues used growth assays that quantified inclusion area, chlamydial genome copy number and infectious progeny to show that a *C. muridarum* strain cured of its plasmid had reduced growth compared to its parental strain (normalization to the number of host cells would have improved the accuracy of strain comparisons). By measuring growth at multiple timepoints in the intracellular infection, they demonstrated that the magnitude of the growth defect varied by timepoint. For example the plasmidless strain produced 2- to 10-fold fewer progeny depending on the timepoint when EBs were harvested (for this and other assays, it would have been helpful to have a supplementary table with the numbers, which could only be estimated from the graphs). This variation by timepoint is not too surprising because of the dynamic nature of the chlamydial developmental cycle – the two strains are each running their own race – but is new and informative because almost all other published studies have used a single timepoint to measure chlamydial growth defects. The growth defects of the plasmid-cured strain stand in contrast to a study by O’Connell and Darville ^1^, which reported no growth difference between plasmid-cured and plasmid-bearing *C. muridarum* strains. The reason for this discrepancy is unresolved.

 What is the utility of this more detailed timepoint analysis? There are hints that the plasmidless strain has a delayed developmental cycle but the article refrained from this level of interpretation. There would be an important difference, particularly with regards to the underlying mechanism, if the growth deficit was due to a reduction or to a delay, or some combination. Inclusion size was smaller with the plasmidless strain but eventually caught up (it would have been good to report the number of inclusions too). However chlamydial genome copies and progeny appeared to show a 4-8 hour delay. Left unmeasured was whether either growth parameter eventually reached the same level as the plasmid-bearing strain. For starters it would be useful to know whether lysis of the plasmidless strain occurred later, which would justify measurements at additional later timepoints.

 The authors’ one step growth curve for infectious progeny is useful for understanding many other studies that have attributed chlamydial growth alterations to chlamydial mutations, inhibitors or depletion of specific host proteins. As in this case, a 5- to 10-fold decrease at a single timepoint is more than likely to be real. However, this study shows that a smaller reduction in the 2-fold range from a single timepoint should be considered with some skepticism because it may not be representative of the overall intracellular infection. In addition, a 2-fold difference is in the range of experimental variation (were the error bars for later time points in Fig. 3 really that small between the two replicates?). Measurements at multiple timepoints would then help to determine the overall magnitude of the chlamydial growth defect and whether it is transient and disappears at later timepoints.

 Overall the study was well performed and will make the *Chlamydia* research community aware of the limitations of measuring growth defects at a single timepoint in the infection. The authors make a compelling case for a role of the plasmid in chlamydial growth because the plasmidless strain produced a large growth defect and contained no other sequence alterations compared to the parental strain. Furthermore there was convincing complementation data showing that reintroduction of the plasmid restored chlamydial genome copy number. This study did not address the mechanism by which the plasmid affects growth, but it is known that a plasmid gene pgp4 encodes a transcriptional regulator of plasmid and chromosomal virulence factors. The conclusions of this article were limited. For example, the bull’s eye inclusion phenotype and the growth deficit were described and measured but there was little explanation or speculation of their causes.

We have read this submission. We believe that we have an appropriate level of expertise to confirm that it is of an acceptable scientific standard.

## References

[1] O'ConnellCMIngallsRRAndrewsCWScurlockAMDarvilleT: Plasmid-deficient Chlamydia muridarum fail to induce immune pathology and protect against oviduct disease. *J Immunol*.2007;179(6) :4027-34 1778584110.4049/jimmunol.179.6.4027

# Peer review: Referee response for version 1

**DOI:** 10.21956/wellcomeopenres.15117.r31733

**Published:** 2018-03-23

**Authors:** Ted Hackstadt

**Affiliations:** 1 Host-parasite Interactions Section, Laboratory of Intracellular Parasites, National Institute of Allergy and Infectious Diseases (NIAID), National Institutes of Health (NIH), Hamilton, MT, USA

Chlamydia are obligate intracellular pathogens that have only recently become amenable to genetic manipulation.  Despite the breakthroughs in recombinant DNA technologies, chlamydiae can be difficult due to their obligate intracellular nature and host specificity, which limits the utility of animal model systems in the study of the human pathogen, *C. trachomatis*.  Skilton *et al *have initiated studies of the murine pathogen *C. muridarum* to examine the role of the endogenous plasmid in pathogenesis.  Here they present a very basic but thorough characterization of plasmid-bearing and plasmid-free *C. muridarum* in cell culture.  Plaque phenotype, rates of progeny formation, and genome equivalents all differ in the plasmid-free strain and the wild type phenotype is restored upon complementation with a newly described shuttle plasmid.  This background information is useful and the shuttle vector system for *C. muridarum* will be valuable for studies of a murine chlamydia strain in mouse models.

I have read this submission. I believe that I have an appropriate level of expertise to confirm that it is of an acceptable scientific standard.
